# Recreational Genetic Databases, Artificial Intelligence, and Forensic Genetics: Technical Advances, Legal Challenges, and Bioethical Perspectives

**DOI:** 10.3390/genes17070730

**Published:** 2026-06-24

**Authors:** Stéphane Sauvagère, Marine Bougerie, Francis Hermitte, Sylvain Hubac, Philippe Manivet, Sabine Kheris, Valérie Duby, Ninon Boissonneau, Christian Siatka

**Affiliations:** 1 Ecole de l’ADN, 19 Grand Rue, 30000 Nîmes, France; sauvagere@ecole-adn.fr (S.S.); sylvain.hubac@gendarmerie.interieur.gouv.fr (S.H.); 2 Institut de Recherche Criminelle de la Gendarmerie Nationale (IRCGN), 95000 Cergy-Pontoise, France; marine.bougerie@gendarmerie.interieur.gouv.fr (M.B.); francis.hermitte@gendarmerie.interieur.gouv.fr (F.H.); 3 Equipe de Recherche sur les Relations Matrice Extracellulaire-Cellules, ERRMECe (EA1391), Groupe Matrice Extracellulaire et Physiopathologie (MECuP), Institut des Matériaux, I-MAT (FD4122), CY Cergy Paris Université, 1 Rue Descartes, 95000 Neuville-sur-Oise, France; 4 UPR-CHROME, Faculté des Sciences Place Gabriel Péri, 30000 Nîmes, France; ninon.boissonneau@etudiant.unimes.fr; 5 Section de Recherches de Marseille—PACA, Caserne Donadieu, 171 Avenue de Toulon, 13010 Marseille, France; 6 AP-HP, GHU, Paris Nord, INTERAXIOME—Service Biologie Médicale Intégrative, Centre de Ressources Biologiques Biobank Lariboisière-Saint Louis (BB-0033-00064), Hôpital Lariboisière, 75475 Paris, France; philippe.manivet@aphp.fr; 7 INSERM U1141 NeuroDiderot, Université de Paris Cité, 75010 Paris, France; 8 IHU Institut Robert Debré du Cerveau de l’Enfant, Hôpital Robert Debré, 75019 Paris, France; 9 Pôle National des Crimes Sériels ou Non Élucidés (PCSNE), Tribunal Judiciaire de Nanterre, 179-191 Avenue Joliot-Curie, 92020 Nanterre, France; sabine.kheris@justice.fr (S.K.); valerie.duby@justice.fr (V.D.)

**Keywords:** direct-to-consumer genetics, investigative genetic genealogy, forensic genetics, artificial intelligence, SNP, STR, DNA phenotyping, kinship analysis, GDPR, bioethics

## Abstract

Background/Objectives: The expansion of direct-to-consumer (DTC) genetic testing has generated civilian genomic databases containing tens of millions of profiles, some of which may be available, under specific conditions, for criminal investigations. Meanwhile, artificial intelligence (AI) is reshaping forensic genetics through applications such as kinship inference, DNA mixture deconvolution, probabilistic phenotyping, and the prioritization of investigative leads. This review examines the scientific, legal, and ethical implications of the convergence between DTC genetic databases, forensic investigative genetic genealogy (FIGG), and AI-assisted forensic analysis. Methods: This article presents a multidisciplinary narrative review at the intersection of forensic genomics, FIGG, artificial intelligence, genomic data governance, and bioethics, with particular attention to French, European, and international regulatory frameworks. Results: Six major dimensions structure the field: (i) the current state of forensic genomic technologies, including STRs, SNPs, and next-generation sequencing; (ii) the contribution of AI to forensic genetics and FIGG; (iii) the governance of large-scale genomic data; (iv) regulatory fragmentation across jurisdictions; (v) the principal bioethical tensions raised by the forensic use of DTC genetic databases; and (vi) future governance needs and operational recommendations. Across these dimensions, three findings emerge. First, genealogical matches and AI-supported outputs should be understood primarily as investigative leads rather than autonomous judicial evidence. Second, the relational nature of genomic data exposes non-consenting relatives to potential forensic scrutiny, thereby challenging traditional models of individual consent and privacy. Third, the absence of harmonized standards for validation, transparency, and oversight remains a major obstacle to legal certainty, judicial admissibility, and public legitimacy. Conclusions: The forensic use of DTC genetic databases should not be understood as a purely technical extension of conventional DNA profiling. It reflects a broader transformation in the relationship between genomic knowledge, criminal investigation, and fundamental rights. Its long-term legitimacy and operational viability will depend on the combined strength of scientific reliability, legal proportionality, ethical safeguards, and meaningful democratic oversight.

## 1. Introduction

This review was developed through a transdisciplinary expert-driven approach bringing together specialists from forensic investigations, forensic genetics, law, bioethics, biomedical research, and clinical practice. The objective was to integrate complementary scientific, legal, ethical, and operational perspectives into a unified framework of analysis.

The originality of this work stems from the convergence of these diverse fields of expertise. Each contributor provided a discipline-specific perspective supported by the scientific, legal, ethical, and technical references considered most relevant within their area of competence. The resulting manuscript therefore offers a comprehensive and integrative analysis of the challenges, opportunities, and implications associated with the subject.

Direct-to-consumer (DTC) genetic testing has transformed access to genomic information by enabling millions of individuals to explore their ancestry, identify biological relatives, and construct family trees outside medical or research settings. A key observation concerning the regulation of DTC genetic testing is the lack of regulatory harmonisation, as legal frameworks differ significantly across jurisdictions and remain non-existent in many countries [1]. In parallel, these commercial services have generated unprecedented civilian genomic databases that are increasingly relevant beyond their original recreational purpose. In some jurisdictions, and under specific legal and technical conditions, such databases have begun to be used in criminal investigations, specifically through forensic investigative genetic genealogy (FIGG), to identify suspects in serious crimes or to assist in the identification of unknown human remains [2]. Clinical genetic testing, by contrast, is intended to address health-related questions within a specifically regulated diagnostic and medical framework and is therefore not considered in this review.

The growing forensic relevance of these databases coincides with another major development: the increasing integration of artificial intelligence into forensic genetics. AI-based tools are now being explored or deployed to prioritize genealogical leads, improve kinship inference, assist with DNA mixture deconvolution or detect male contribution, and support probabilistic phenotype prediction. While these technologies broaden investigative capabilities, they also challenge existing legal categories, ethical safeguards, and evidentiary standards [3].

This review examines the convergence of DTC genetic databases, forensic genetics, and AI from a multidisciplinary perspective at the intersection of genomics, law, and bioethics. Its purpose is to clarify key concepts, assess current technological and methodological capacities, identify legal and ethical conflicts, and propose practical recommendations for future governance. The added value of this review lies in its French and European governance perspective: it does not merely summarize the technical development of FIGG, DTC genetic databases, and AI-assisted forensic genetics, but examines how their convergence challenges legal admissibility, data-protection frameworks, bioethical safeguards, and operational decision-making in jurisdictions where these practices remain incompletely regulated.

### 1.1. Definitions and Conceptual Distinctions

Recreational genetics, also referred to as consumer or direct-to-consumer genetics, encompasses commercial services that offer individuals access to DNA analysis for non-medical purposes, primarily ancestry inference, genealogical reconstruction, and the identification of biological relatives. Since the mid-2000s, companies such as Ancestry, 23andMe, MyHeritage, and FamilyTreeDNA have developed large-scale commercial platforms built around these services. The resulting databases now contain tens of millions of civilian genetic profiles, giving them a population coverage that, in some settings, exceeds that of institutional forensic databases [4].

Three categories should be clearly distinguished, although they are often conflated in the literature. First, DTC genetic databases are genomic datasets collected in a commercial context, without an original law-enforcement purpose, from consumers who consented to terms and conditions that are frequently lengthy and difficult to interpret. Second, genetic genealogy refers to the use of genomic tools to reconstruct biological relatedness, whether in civil or judicial contexts. Third, forensic genetics refers to the scientific and legal use of DNA analysis in criminal or civil investigations [5].

Forensic Investigative Genetic Genealogy (FIGG) lies at the intersection of these three categories. It applies methods from genetic genealogy to criminal investigations or human identification efforts, often relying, though not exclusively, on DTC or publicly accessible civilian databases. The Golden State Killer case remains the most widely cited example of this approach, illustrating, from relevant evidence collected on the crime scene (sperm), how distant kinship matching combined with genealogical reconstruction can generate investigative leads in cases that had remained unresolved for decades [6,7].

### 1.2. Use Cases, Benefits, and Risks

The main use cases of FIGG can be grouped into three broad categories. The first, historically benefiting the most from the media, concerns the identification of suspected perpetrators of serious crimes, such as homicide, sexual assault, and long-unsolved cold cases, when no match is obtained from institutional forensic databases [8]. The second concerns the identification of unknown victims, particularly in the context of mass disasters, armed conflict, migration, or missing persons investigations. The third, more recent application involves the exoneration of wrongly convicted individuals through broader genetic comparison strategies [9]. Recent compilations of FIGG-related casework suggest that its practical applications are more evenly divided between offender identification and human identification than public attention alone would suggest. According to a 2025 project dataset reporting 1435 FIGG-associated identifications, 52% of cases concerned the identification of perpetrators, while 47% involved the identification of victims or human remains [10,11]. The latter category appears to be increasing, particularly in the context of disaster victim identification and the analysis of unidentified remains. The use of FIGG is also expanding in Europe. The Netherlands, Sweden, and Denmark, have recently legalized and regulated this technique on the basis of criteria that increasingly appear to reflect an emerging international consensus: limitation to the most serious crimes against persons, subsidiarity, prior authorization by a judicial authority, restriction to private DNA databases ensuring a minimum level of privacy protection, and user consent to the use of their data in the context of judicial proceedings [12,13,14].

The documented benefits are substantial. These include the resolution of decades-old cases, improved investigative efficiency, and the delivery of answers to victims and families. Yet these gains include systemic risks that require scrutiny.

From a privacy standpoint, the core issue is the non-consensual implication of genetic relatives. When one individual uploads their DNA data to a DTC platform, they indirectly reveal information not only about themselves but also about their biological relatives, parents, siblings, children, and cousins, who may never have consented to genetic testing or forensic use. This relational nature of genomic data fundamentally complicates traditional models of individual consent [15].

Another major concern is structural bias. DTC genetic databases disproportionately represent individuals of European American ancestry, resulting in unequal performance across populations. This affects both suspect identification and victim identification. Some groups may be less likely to generate useful kinship matches, while others may face different forms of forensic under-representation or overexposure depending on the database structure and investigative context. These asymmetries raise important concerns about fairness, discrimination, and distributive justice in forensic practice [16].

The forensic use of DTC genetic databases is therefore not merely a technical matter. It reflects a deeper tension between the public interest in solving serious crimes or identifying unknown human remains and the protection of fundamental rights, including privacy, autonomy, equality, and informed consent.

### 1.3. General Legal Background

The legal framework governing FIGG remains fragmented and rapidly evolving. In Europe, the General Data Protection Regulation (GDPR) classifies genetic data as a special category of personal data subject to enhanced protection. In parallel, the Law Enforcement Directive regulates the processing of personal data for criminal justice purposes, yet its articulation with the GDPR in the context of DTC genetic databases remains contested. In France, the relevant legal landscape includes bioethics legislation, criminal procedure rules governing forensic DNA databases, and national data protection law [17].

In the United States, the absence of a unified federal framework on genetic privacy has produced a patchwork regulatory environment. Existing legislation addresses certain forms of genetic discrimination but does not comprehensively govern the forensic use of DTC genetic databases. As a result, practical guidance has often emerged from agency-level policies, company terms of use, and state-based initiatives rather than from a coherent statutory model. At the international level, the lack of harmonized and binding instruments creates significant risks of legal inconsistency, jurisdiction shopping, and uneven protection of individuals’ rights [18].

## 2. Current State of Forensic Genomic Technologies

### 2.1. STRs, Sequencing, and SNPs: Current Status and Performance

#### 2.1.1. STR Markers and the Classical Forensic Paradigm

Short tandem repeat (STR) markers have formed the backbone of institutional forensic genetics since the 1990s. An STR profile is generated by genotyping a limited number of polymorphic loci, typically those used within CODIS in the United States or within the European Standard Set (ESS), thereby producing a statistically near-unique identifier for direct human identification. Their analytical robustness and reliability, inter-laboratory reproducibility, and long-standing judicial acceptance have made STRs the reference standard for direct comparisons between an unknown biological trace and a suspect or offender reference sample [19].

However, institutional STR profiles are structurally incompatible with the data generated by DTC genetic databases. DTC platforms generally rely on SNP microarrays or next-generation sequencing (NGS)-based approaches that generate single-nucleotide polymorphism (SNP) data rather than STR profiles. This difference in marker architecture remains one of the major technical barriers to interoperability between institutional forensic systems and civilian DTC genetic databases [20,21].

#### 2.1.2. SNPs and DTC Genomic Arrays

Standard DTC genetic tests are generally based on DNA microarrays interrogating approximately 500,000 to 900,000 SNPs distributed across the genome. These SNPs are selected for their informativeness in ancestry inference and kinship analysis rather than for maximal individual discrimination, as is the case for STRs. Their forensic value in IGG lies primarily in their ability to reconstruct haplotypes and estimate degrees of relatedness through shared segments that are identical by descent (IBD) [22].

In this context, the core metric is not an exact profile match, but the amount of shared IBD DNA, usually expressed in centimorgans (cM). Shared values around 3400 cM are consistent with parent–child or full-sibling relationships, whereas values in the range of roughly 850 to 1400 cM may indicate second-degree relationships, such as avuncular or grandparent–grandchild relationships. Below approximately 200 cM, kinship inference becomes substantially more ambiguous and generally requires more advanced probabilistic interpretation. Investigative genetic genealogy therefore relies on partial and probabilistic kinship signals, rather than on the direct one-to-one individualization paradigm that characterizes conventional STR-based forensic comparison [23,24,25].

#### 2.1.3. Next-Generation Sequencing in Forensic Contexts

Massively parallel sequencing (MPS), also referred to as next-generation sequencing (NGS), has opened important new perspectives in forensic genomics. It enables the recovery of information from highly degraded samples, including old skeletal remains, compromised hair samples, and challenging trace materials [26]. It also allows the simultaneous interrogation of multiple marker classes, including autosomal STRs, SNPs, Y-chromosome markers, mitochondrial DNA, and markers associated with externally visible characteristics (EVCs) [27].

Commercial platforms such as ForenSeq and Precision ID exemplify this technological convergence. Nevertheless, forensic implementation of NGS still faces several challenges, including relatively high costs, incomplete analytical standardization, the absence of universally accepted minimum quality thresholds for judicially reliable use, and the limited availability of reference panels adequately representing global population diversity [20].

### 2.2. Analytical Pipelines, Quality Control, and Limitations

#### 2.2.1. Quality Control and Quantification

The validity of forensic genomic analysis depends on rigorous quality control procedures across the entire analytical workflow. DNA quantification, typically performed using quantitative PCR (qPCR) or droplet digital PCR (ddPCR), guides the choice of amplification strategy and helps anticipate allele imbalance and related stochastic effects [28,29]. Sequencing quality metrics, such as mean coverage, coverage uniformity, and the proportion of Q30 bases, should be documented systematically and evaluated against predefined analytical thresholds. The lack of harmonized quality standards across laboratories remains a major source of variability. This variability affects both inter-laboratory comparability and the judicial admissibility of genomic evidence, especially in complex or low-template cases [30].

#### 2.2.2. DNA Mixtures

DNA mixture deconvolution remains one of the most technically demanding areas of forensic genetics. Such mixtures are common in sexual assault cases and in crime scenes involving multiple biological contributors. Two-person mixtures can often be interpreted using probabilistic genotyping software such as STRmix, TrueAllele, Genemapper or EuroForMix, which model allele distributions and calculate likelihood ratios to assess the probability that a given individual contributed to the mixture [31]. Performance declines, however, as the number of contributors increases and the quality of DNA decreases. In mixtures involving three or more individuals, uncertainty rises substantially and interpretive complexity increases accordingly. Dror and Hampikian have extensively documented the subjectivity and interpretative biases associated with DNA mixtures, illustrating the complexity of mixed DNA profile interpretation [32]. Under such conditions, transparent reporting of uncertainty, model assumptions, and confidence intervals becomes essential for responsible expert communication [33].

#### 2.2.3. Drop-Out, Drop-In, and Analytical Noise

Allelic drop-out, the stochastic failure to detect one allele during amplification, and drop-in, the appearance of spurious allelic signals, are recurrent sources of error in low-template or degraded samples. Their frequency increases as DNA quantity and quality decline. In STR-based analyses, stutter peaks represent an additional source of interpretive difficulty, particularly in complex mixtures, because they can be mistaken for minor contributor alleles [34]. Probabilistic modeling of these artifacts has improved substantially over the past decade. Even so, such models typically require laboratory-specific calibration parameters, which limits portability and complicates broader standardization across forensic systems [35].

#### 2.2.4. Limits of Long-Range Investigative Genetic Genealogy

Long-range IGG, which attempts to identify an unknown individual through distant relatives sharing a common ancestor four to six generations back, faces intrinsic statistical limitations [36]. Over successive generations, IBD segments become shorter and more fragmented, making distant kinship inference increasingly uncertain. For relationships beyond roughly the fourth-cousin level, where shared DNA may fall below 100 cM, statistical noise becomes a dominant issue and false-positive kinship signals become more likely [37].

This limitation is particularly important in forensic practice, because genealogical inference at such distances may generate plausible yet ultimately incorrect investigative leads [38]. It therefore reinforces the need for independent confirmation and careful judicial scrutiny before any genealogical lead is transformed into evidentiary material.

### 2.3. Technology Harmonization and Standardization

Interoperability between DTC genetic databases and institutional forensic systems requires substantial standardization efforts. Several dimensions are involved, including file formats, SNP panel compatibility, overlap between commercial and forensic marker sets, inter-laboratory quality control procedures, and validation frameworks for interpretation software. At present, no international standard mandates a common format or a minimum quality threshold for DTC genetic databases used in forensic investigations. In the United States, SWGDAM has issued guidance documents, while in Europe ENFSI has contributed recommendations. However, these initiatives remain non-binding and unevenly implemented [39]. A more structured harmonization effort, ideally organized through an international scientific consortium bringing together DTC industry stakeholders, forensic laboratories, regulators, and academic experts, appears necessary to ensure both technical reliability and judicial acceptability at scale. Existing initiatives, including developments related to Prüm II in Europe, represent important progress for STR-based exchanges between Member States, but they do not yet adequately address the specific challenges posed by SNP data originating from DTC genetic databases [12].

## 3. Artificial Intelligence in Forensic Genetics

### 3.1. Use Cases of Artificial Intelligence in Investigative Genetic Genealogy

At this stage, not all AI applications discussed in forensic genetics have reached the same level of maturity. Some are already integrated, at least partially, into applied forensic workflows, whereas others remain experimental or proof-of-concept and should not be presented as operationally established [40]. To avoid conflating promise with practice, the following sections distinguish between tools supported by validation and real-world use, and those that still require substantial technical, forensic, and legal evaluation before routine implementation can be considered [41].

#### 3.1.1. DNA Mixture Deconvolution

Artificial intelligence, and more specifically deep learning approaches, offers promising perspectives for improving the deconvolution of complex DNA mixtures. Transformer-based architectures and convolutional neural networks trained on synthetic mixtures generated in silico have shown performance gains over conventional probabilistic approaches in scenarios involving three or more contributors under conditions of moderate degradation [42]. Some recent studies have reported reduced error rates in simulated mixture scenarios relative to more traditional methods [43]. However, these results still require validation on real forensic datasets and under authentic casework conditions before their evidentiary robustness and reliability can be considered established [44]. This caution is even more necessary for SNP-based mixture deconvolution than for STR-based interpretation. Whereas STR mixture analysis benefits from established forensic practice, better-characterized artefactual patterns, and comparatively more mature validation frameworks, SNP mixture analysis remains more difficult to standardize because of marker panel heterogeneity, variable sequencing performance, more complex contributor modeling, and the current lack of broadly accepted analytical thresholds and inter-laboratory benchmarks [20].

#### 3.1.2. Probabilistic DNA Phenotyping

DNA phenotyping, the inference of physical traits from the genomic profile of an unknown individual, represents one of the most sensitive forensic applications of AI. Predictive models for eye color, hair color, skin pigmentation, aspects of facial morphology, or biological age have been developed or commercialized by pioneer companies such as Parabon NanoLabs, the VISAGE Consortium, and Identitas [45]. These tools are intended to assist investigations when conventional leads are lacking or insufficient. The limitations of these approaches are substantial. First, predictive accuracy varies specifically across traits: eye color prediction may achieve relatively high performance, whereas facial morphology and fine-scale biogeographical ancestry remain much more uncertain. Second, the use of such genetic composites in investigative contexts may introduce cognitive bias among investigators or reinforce discriminatory effects against vulnerable groups. Several European jurisdictions have therefore restricted or prohibited parts of this practice, particularly where it is considered incompatible with proportionality requirements or the presumption of innocence [46].

#### 3.1.3. Long-Range Kinship Inference

The detection and quantification of IBD segments shared between distantly related individuals lies at the technical core of IGG. Algorithms such as GERMLINE, IBDseq, RefinedIBD, and hap-IBD were initially developed for population-genetic and epidemiological research before being adapted to forensic applications [47]. More recently, machine-learning approaches, including gradient boosting methods and graph neural networks that model genealogical relationships as graph structures, have improved the estimation of relatedness in ambiguous overlap zones, particularly in the approximate 50–200 cM range [48]. The central challenge is to reduce both false positives, corresponding to over-inference of kinship, and false negatives, corresponding to missed genuine matches.

### 3.2. Standardization of Forensic AI

The performance of any AI model depends fundamentally on the representativeness and quality of its training data. In forensic genetics, this requirement is constrained by several structural limitations. First, labeled datasets available for training are frequently derived from specific populations, most often individuals of European American ancestry, thereby producing biased models that underperform in other groups [49]. Second, authentic forensic datasets are scarce, fragmented, and often subject to confidentiality restrictions, which promotes reliance on synthetic datasets whose realism and representativeness must themselves be critically assessed. Third, socio-economic and racial biases embedded in criminal justice systems may become encoded in training data and subsequently amplified by model outputs. The European AI Act classifies AI systems used in law enforcement contexts among high-risk systems and subjects them to enhanced requirements regarding transparency, documentation, traceability, and ex ante conformity assessment [50]. These requirements are especially difficult to satisfy for deep learning architectures, which are often criticized for their black-box character. Post hoc explainability approaches, such as SHAP, LIME, or attention-based visualization techniques, can provide partial insight into model behavior, but they do not fundamentally resolve the problem of algorithmic opacity.

The deployment and maintenance of AI models in forensic settings require rigorous machine learning operations practices, including model versioning, prediction logging, performance drift monitoring, requalification protocols, and comprehensive documentation of training parameters [51]. Standardized performance metrics, such as sensitivity, specificity, area under the ROC curve, and acceptable false-positive rates, should be defined in light of the intended use context and the relevant legal threshold for tolerable error. In the absence of a unified technical framework, however, vendors often define their own metrics and validation criteria, making cross-system comparisons difficult and weakening judicial acceptability [52]. Full traceability of the analytical chain, from the original sample to the final forensic conclusion, including each algorithmic transformation, should therefore be regarded as a core requirement for reproducibility and adversarial scrutiny.

### 3.3. Investigative Lead Versus Judicial Evidence: Preventing a Fundamental Conceptual Confusion

One of the main epistemological and procedural risks associated with FIGG and AI-assisted forensic tools lies in the confusion between an investigative lead and judicial evidence. This confusion, common in media discourse and sometimes reinforced by technologically centered presentations, must be explicitly rejected. Under current scientific knowledge and operational practice, a genealogical correspondence derived from a DTC genetic database does not, by itself, constitute proof of criminal responsibility. At most, it provides an orienting signal, that is, a probabilistic indication that may help generate, narrow, or rank investigative hypotheses [53]. It should be noted that ancestry inference provided by DTC genetic testing services is often partially based on self-reported population origin data. This reliance on self-declared information introduces a potential source of reporting bias that may affect the robustness and interpretation of ancestry assignments.

From a scientific standpoint, the logic of FIGG is indirect. Unlike a conventional STR comparison between an evidentiary trace and an individualized reference sample, genealogical searching does not produce direct identification. Rather, it detects a level of shared DNA compatible with a more or less distant biological relationship. The relevant metric is not strict profile identity, but the sharing of IBD segments, the interpretation of which becomes increasingly uncertain as relatedness becomes more remote [9,45].

The same reservation applies, a fortiori, to AI tools used in support of FIGG. Whether the system is designed for kinship scoring, automated lead prioritization, probabilistic phenotyping, or algorithmic candidate ranking, its function is not to prove, but to reduce the search space. Its normative product must therefore be defined with precision: not as autonomous evidence, but as an instrument for generating and ordering investigative hypotheses [54].

Procedurally, this distinction has a decisive implication: judicial confirmation must occur at a later stage, through independent, validated, and contestable techniques. In forensic genetics, such confirmation typically relies on a lawfully obtained reference sample followed by a standardized analytical comparison, usually STR-based and, where appropriate, supplemented by other material or contextual evidence. Until this independent verification has been completed, information derived from a DTC genetic database or from an algorithmic model should not be presented as evidence, but as an investigative lead [55].

This distinction is not merely terminological. It determines the legal regime applicable to the information produced. To classify a genealogical correspondence as evidence would be to assign it an intrinsic probative force that it does not possess, thereby risking circumvention of the classic guarantees of a fair trial: intelligibility of expert reasoning, meaningful opportunity for challenge, access to relevant parameters, scrutiny of methodological reliability, and adversarial assessment of evidentiary weight [12].

A further procedural risk is cognitive lock-in. Once a genealogical or algorithmic hypothesis appears particularly salient, investigators may be inclined to privilege that target prematurely at the expense of competing hypotheses. The more sophisticated or “scientific” the tool appears, the greater the risk of excessive deference to its output. Yet neither a kinship probability, nor a phenotypic projection, nor an algorithmic ranking is self-sufficient. All depend on modeling assumptions, reference datasets, calibration parameters, and sometimes proprietary systems whose transparency and explainability remain incomplete [6,56].

A disciplined qualification framework is therefore required. The legally appropriate sequence may be summarized as follows: unknown biological trace → genealogical or algorithmic exploratory analysis → formulation of a nominative or familial hypothesis → independent verification by validated forensic methods → adversarial assessment of final probative value. Only at the end of this sequence may an initially indicatory element contribute to proof [57]. This expanded sequence is important because FIGG does not operate as a direct inferential shortcut from database hit to evidentiary conclusion. Between the initial genealogical signal and the final forensic confirmation, there is often a substantial operational phase involving family tree reconstruction, archival and civil-record research, assessment of demographic and geographic plausibility, and conventional investigative work. In practice, the genealogical lead must be progressively tested against real-world information before any reference sampling is even contemplated [58]. This intermediate investigative stage is essential, both to reduce the risk of misidentification and to reflect the actual workflow of FIGG in operational settings.

Ultimately, IGG and AI tools applied to forensic genetics should be understood, in legal terms, as technologies of investigative orientation rather than autonomous mechanisms of proof. Their legitimacy depends precisely on maintaining that boundary.

## 4. Large-Scale Data Management in Forensic Genetics

### 4.1. Nature and Taxonomy of Genomic Data

The data generated in the context of FIGG are highly heterogeneous and therefore require precise categorization in order to support appropriate governance. A conventional distinction is made between coding genomic data, variants located in exonic regions that may influence biological or pathological phenotypes, and non-coding data, historically considered functionally silent [59]. Although this distinction remains relevant in molecular biology and retains legal significance in several regulatory instruments, including the Oviedo Convention, its practical value has progressively diminished as regulatory and functional roles have increasingly been identified within non-coding regions [60].

This classification must be complemented by other categories that are particularly relevant in forensic genetics. These include mitochondrial haplotypes, transmitted through the maternal line and useful for matrilineal identification, as well as Y-chromosome haplotypes, transmitted through the paternal line and relevant to patrilineal investigation. Such data have a collective dimension, since they provide information not only about the tested individual but also about a broader lineage [61]. This shared familial significance reinforces their particular sensitivity from both ethical and legal perspectives.

In addition, associated metadata, including signal quality, geographical origin of the sample, date of collection, laboratory processing history, and chain-of-custody information, must themselves be regarded as sensitive data [59]. Even where genomic datasets are nominally anonymized, these metadata may enable re-identification when combined with external information sources.

### 4.2. Governance Architectures

#### 4.2.1. Anonymization, Pseudonymization, and Data Minimization

True anonymization of genomic data is extraordinarily difficult and may be impossible to guarantee over the long term. Foundational studies have shown that apparently anonymized genomic profiles can be re-identified with high probability through linkage with other publicly or semi-publicly available resources, including genealogical registries, medical datasets, and social media data [62]. In practice, pseudonymization provides only partial protection, since the process remains reversible under certain conditions and therefore requires additional technical and organizational safeguards [63].

The principle of data minimization, enshrined in Article 5(1)(c) of the GDPR, requires that only data strictly necessary for the declared purpose be collected and processed [64]. The secondary use of genetic data generated through direct-to-consumer testing raises important legal and ethical considerations. Under data protection frameworks such as the GDPR, the processing of genetic data must be based on a clearly identified legal basis and comply with the principle of purpose limitation, whereby data collected for one purpose should not be reused for unrelated objectives without appropriate safeguards [65]. Although scientific research may constitute a legitimate basis for further processing under specific conditions, such reuse generally requires transparent information and, in many cases, explicit consent from the data subject. Given the sensitive nature of genetic information and its potential implications for both individuals and their biological relatives, robust governance mechanisms are essential to ensure lawful, ethical, and responsible secondary use of these data. In the context of IGG, this principle calls for a careful definition of which genomic markers are genuinely necessary for investigative purposes and argues against opportunistic reuse of data collected for other objectives.

#### 4.2.2. Tiered Access and Logging

Access to DTC genetic databases in judicial investigations should be governed through a tiered model calibrated to both the seriousness of the offense and the subsidiarity of the investigative measure [12]. A three-level framework may be envisaged: restricted access to IBD-based kinship information only; expanded access to selected phenotypic inferences, subject to prior judicial authorization; and access to raw genomic data only in exceptional circumstances and under strict independent supervision [66]. Such a tiered model should apply not only to the categories of data that may be accessed, but also to the categories of actors authorized to access them, with clearly differentiated roles for forensic scientists, investigators, and judicial authorities according to operational need, legal competence, and the sensitivity of the information concerned. Comprehensive logging of every access event is an essential requirement of traceability and accountability. Audit logs should record, at a minimum, the identity of the requesting party, date and time of access, categories of data consulted, legal basis invoked, and outcome of the query [67]. These logs must themselves be protected against alteration and retained for a sufficient period to allow retrospective oversight.

#### 4.2.3. Cross-Border Data Flows and Jurisdictional Fragmentation

A further governance challenge arises from the transnational nature of many DTC genetic databases [68]. Data may be collected in one country, stored in another, processed in a third, and queried by judicial authorities in a fourth. This fragmentation creates significant uncertainty regarding the applicable legal regime, the validity of user consent, the adequacy of transfer safeguards, and the enforceability of defense rights. For this reason, any governance architecture for IGG should explicitly address jurisdictional allocation, lawful transfer mechanisms, and minimum guarantees applicable regardless of the storage location of the database [69]. Without such safeguards, the risk is not only legal inconsistency but also regulatory arbitrage.

Within the European Union, these issues are particularly relevant because genetic data constitute a special category of personal data under Article 9 of the General Data Protection Regulation (GDPR). Cross-border transfers of such data are subject to the requirements established in Chapter V of the GDPR, including adequacy decisions, standard contractual clauses, binding corporate rules, or other legally recognised transfer mechanisms. However, the practical application of these safeguards remains complex when forensic investigations involve private DTC databases established outside the European Economic Area [64,68].

Additional challenges arise from the coexistence of different regulatory regimes. While the GDPR governs personal data processing in the civilian sector, law enforcement activities are principally regulated through Directive (EU) 2016/680 (Law Enforcement Directive). The interaction between these instruments remains insufficiently clarified in the context of forensic investigative genetic genealogy, particularly when genetic information originally collected for recreational purposes is subsequently accessed for criminal investigations. Consequently, future governance frameworks for IGG should explicitly define jurisdictional responsibilities, lawful transfer mechanisms, accountability obligations, and minimum procedural safeguards applicable irrespective of the geographical location of data storage. Such safeguards are necessary not only to ensure legal certainty and regulatory consistency but also to preserve public trust and the legitimacy of forensic uses of civilian genomic databases. Regulatory fragmentation may otherwise create opportunities for forum shopping and regulatory arbitrage, potentially weakening the protection of fundamental rights.

### 4.3. Storage and Governance of Biological Samples

Beyond digital data, the management of physical biological samples raises distinct governance questions. Retention periods should be proportionate to the investigative purpose and to the nature of the case. In resolved cases, rapid destruction of superfluous samples is generally preferable in order to reduce the risk of secondary or unauthorized use [70]. In unresolved cases, longer retention may be justified, provided that legal authority, scientific utility, and storage conditions are clearly documented [71].

Cold-chain requirements, freeze–thaw procedures and their impact on DNA integrity, and protocols for certified destruction or long-term archival storage all require precise technical and legal regulation [72]. Physical traceability is therefore a sine qua non condition for the judicial use of biological evidence. Retention registers, secure labeling systems, documented transfers between laboratories, and controlled access procedures must all form part of a robust chain-of-custody framework.

## 5. Regulatory and Ethical Frameworks

### 5.1. France, Europe, and the International Context

#### 5.1.1. The French Legal Framework

Under French law, genetic identification for judicial purposes is primarily governed by the “Code de procédure pénale”, together with the revised “Loi Informatique et Libertés”. The “Fichier National Automatisé des Empreintes Génétiques” (FNAEG), established in 1998 and progressively expanded thereafter, remains the central institutional tool of forensic genetics in France [73]. Its scope, access conditions, and the rights of registered individuals have generated substantial litigation, including European scrutiny concerning proportionality and retention practices [74].

By contrast, the use of DTC genetic databases in French criminal investigations is not expressly regulated at present, creating a significant legal grey area. CNIL has issued opinions on recreational genetic testing and the protection of genetic data but has not yet articulated a fully developed position and response on their specific forensic exploitation [75]. Likewise, the French National Consultative Ethics Committee for Health and Life Sciences (Comité Consultatif National d’Éthique, CCNE) has emphasized the risks to privacy, autonomy, and informed consent while calling for broader public and legislative reflection [76]. This issue is currently under active discussion in France. In particular, a recent bill before the National Assembly seeks to authorize access to recreational genetic databases for the identification of perpetrators of serious crimes and serious offences, thereby reflecting an emerging legislative effort to reduce the current legal uncertainty surrounding the use of FIGG in criminal investigations [77].

#### 5.1.2. The European Framework

At the European level, the legal environment is shaped by several overlapping instruments. The GDPR classifies genetic data as a special category of personal data subject to enhanced protection, while Directive (EU) 2016/680 regulates personal data processing for criminal justice purposes [78]. The interaction between these two regimes becomes particularly complex when data originally collected in a private commercial context are later accessed, reused, or relied upon for law enforcement purposes. More broadly, the Oviedo Convention and the Charter of Fundamental Rights of the European Union provide foundational principles for the protection of genetic integrity, dignity, privacy, and informational self-determination [79]. The Prüm framework, and its ongoing evolution through Prüm II, organizes the automated exchange of DNA data among Member States. Yet this system was designed around STR profiles and remains technically and conceptually distant from the SNP-based logic of DTC genetic databases. In response to this fragmented regulatory landscape, the Appendix A includes a dedicated overview entitled “European countries without legislation specifically and exclusively dedicated to forensic DNA databases or DNA profiling (as of 1 January 2026)”, listing European jurisdictions in which no specific and exclusive legal framework governing forensic DNA databases or DNA profiling could be identified.

#### 5.1.3. The International Context

Outside Europe, regulatory approaches remain fragmented. In the United States, CODIS constitutes the core institutional forensic DNA infrastructure, while guidance on FIGG has developed through agency policies, provider terms of service, and state-specific legislative initiatives rather than through a single unified federal statute [80]. This has produced a patchwork model in which access conditions, offense thresholds, procedural safeguards, and admissibility concerns may vary significantly across jurisdictions [81].

In low- and middle-income countries, FIGG raises additional concerns, including limited laboratory and bioinformatic capacity, weaker regulatory infrastructures, asymmetrical dependence on foreign technology providers, and the risk that local populations may become data sources without equivalent control over governance or benefit-sharing.

### 5.2. Ethics and Bioethics

#### 5.2.1. Proportionality and Purpose Limitation

The principle of proportionality requires that any interference with fundamental rights resulting from access to DTC genetic databases be justified by a sufficiently weighty public interest and that the means employed be appropriate and necessary to the intended objective. In practice, this principle strongly supports limiting FIGG to the investigation of the most serious offenses. This position is partly reflected in current practice, as access to some DTC genetic databases is already restricted by providers to specific categories of cases, such as violent crimes or the identification of unidentified human remains, sometimes on the basis of explicit opt-in consent by users. Likewise, FIGG is not generally conceived as a tool for minor offenses, but rather for serious criminal cases in which conventional forensic approaches have failed to produce an identification. Closely related is the principle of purpose limitation. Genetic data initially collected for ancestry exploration or family reconstruction should not be repurposed for law-enforcement objectives without careful scrutiny of compatibility, legal basis, and user expectations [82]. Because genomic information is relational rather than purely individual, purpose limitation in this field must address not only the tested person, but also the broader family network indirectly exposed by their participation [83]. However, these practical safeguards remain unevenly defined and inconsistently applied across providers and jurisdictions, which limits their capacity to substitute for a clear and harmonized legal framework.

#### 5.2.2. Consent in DTC Genetic Databases

Consent is arguably the most ethically difficult issue in this field. A liberal view holds that adult users who accepted terms and conditions permitting certain research, public-interest, or law-enforcement uses have provided legally and ethically sufficient consent [84]. A restrictive view argues that such consent is often undermined by major informational asymmetries between providers and consumers, by the length and opacity of contractual documents, and by the practical impossibility of anticipating the future consequences of forensic reuse at the time of agreement [85]. An intermediate position advocates a specific, informed, granular, and revocable consent mechanism for forensic use that is separate from the initial consent to recreational testing. Such an approach does not eliminate all ethical concerns, especially those affecting relatives who did not consent at all, but it better aligns the architecture of consent with the actual sensitivity of the use [86].

#### 5.2.3. Risks of Function Creep, Bias, and Discrimination

The forensic use of genomic data creates several well-documented risks of systemic drift. First, there is a risk of unequal exposure across population groups. Because institutional forensic databases and DTC genetic databases are not demographically balanced in the same way, the probability of identification, exclusion, or investigative attention may vary according to ancestry, geographic origin, and family structure [87]. Second, phenotypic inference from DNA may encourage discriminatory investigative practices if not subject to strict limitations. Third, there is the classic risk of function creep. A technique initially justified for exceptional use in the investigation of the gravest offenses may gradually become normalized, expanded, and redeployed in broader contexts unless legal and institutional safeguards are explicit and enforceable [88,89].

The democratic legitimacy of forensic access to DTC genetic databases depends on cumulative safeguards rather than on technical utility alone. At a minimum, these safeguards should include public transparency regarding actual use cases and outcomes, independent judicial and/or parliamentary oversight, regular publication of aggregated statistics, audit mechanisms conducted by independent bodies, and meaningful procedural rights for affected persons wherever compatible with the needs of criminal investigation [12,90]. Without such safeguards, there is a serious risk of de facto privatization of part of the state’s investigative power. Commercial actors may become gatekeepers of access to genetic information that has major implications for liberty, privacy, and criminal procedure, while operating under incentive structures that do not necessarily coincide with the public interest.

A further bioethical issue deserves explicit treatment: the concept of relational privacy. Unlike many other categories of personal data, genomic data inherently reveal information about biological relatives [91]. The decision of one individual to upload DNA data to a DTC platform may therefore affect the privacy, autonomy, and legal exposure of family members who neither participated nor consented. In the context of IGG, relational privacy implies that governance cannot rely exclusively on the voluntariness of the initial contributor. It must also account for the distributed nature of genetic exposure [8,39]. This requires a more family-aware conception of genomic responsibility.

### 5.3. Open Legal and Societal Questions

Several fundamental questions remain unresolved in current positive law. Can a genetic profile obtained from a foreign DTC genetic database without formal judicial cooperation be treated as procedurally valid in a European criminal proceeding? Who bears responsibility for demonstrating compliance with applicable data-protection requirements: the investigator who queried the database, the provider that granted access, or both jointly?

May an individual be compelled to provide a reference DNA sample solely on the basis of a genealogical correspondence obtained through IGG, despite not being a direct suspect at the outset? [92] Do DTC companies have a legal duty to cooperate with judicial authorities, or merely a policy-based discretion to do so? How should legal systems protect genetically distinctive minorities, including Indigenous populations and endogamous communities, from disproportionate identifiability?

A further unresolved issue concerns remedies. If an individual is identified, investigated, or indirectly exposed through an unlawful or disproportionate use of IGG, what remedies should be available? [5,45] Exclusion of evidence, deletion of data, civil liability, regulatory sanctions, and judicial review all appear relevant, but few legal systems have yet developed a coherent remedial framework tailored to this type of genomic intrusion [93].

## 6. Future Directions and Recommendations

### 6.1. Creating Dedicated Forensic Genetic Databases

The creation of genetic databases specifically designed for forensic use, and clearly distinct from commercial DTC genetic databases, represents a promising avenue for reconciling investigative efficiency with the protection of fundamental rights. Such databases should be built on strict eligibility and governance criteria, including voluntary and informed participation, explicit forensic purpose specification at the time of consent, independent governance by a multidisciplinary body, ex ante oversight by an independent authority, and ex post control through mandatory periodic audits [12]. Their governance model could draw on best practices developed in research biobanking while incorporating the specific procedural and evidentiary constraints of the forensic context. A “forensic-by-design” model of this kind would help address several of the ethical and legal tensions identified in this review.

### 6.2. Interoperability and Open Standards

Interoperability across forensic systems, national, European, and international, remains a prerequisite for effective cooperation. Achieving this goal requires the development and adoption of open standards covering several dimensions: common metadata patterns describing format, quality, provenance, and consent conditions; secure interfaces for the exchange of profiles, matches, and audit information; and shared minimum quality benchmarks for the forensic use of genomic data [93]. An internationally recognized forensic quality reference system would represent a major step forward. Such a framework should cover the full analytical chain, sampling, extraction, quantification, sequencing or genotyping, interpretation, reporting, and long-term traceability, and should address the specific characteristics of SNP-based kinship data used in FIGG [94]. This also calls for the progressive professionalization of FIGG-specific genealogy as a distinct operational expertise, with clearer definition of expected competencies, dedicated training pathways, shared methodological standards, and more explicit oversight of professional practices.

### 6.3. Europol and Interpol Cooperation

Strengthened international cooperation is essential in order to overcome the limitations of purely national approaches in a context of increasingly transnational crime. Europol already has analytical capacities relevant to forensic intelligence, while Interpol coordinates the FIND network, which facilitates exchanges among forensic laboratories in member states [95]. In this context, at the European level, the European Network of Forensic Science Institutes (ENFSI) could be seen as particularly well placed to contribute to the development of FIGG-specific technical guidance [96], while the European Academy of Forensic Science (EAFS) could provide an important forum for scientific exchange and consensus-building. Both could therefore play a particularly important role in the development of dedicated FIGG guidelines, especially regarding technical standards, validation requirements, reporting practices, and inter-laboratory harmonization. These structures could be further developed to include exchange protocols specifically tailored to data originating from DTC genetic databases, provided that such developments are accompanied by explicit safeguard frameworks [97]. The establishment of mixed technical and ethical evaluation units could provide a useful institutional innovation. Such bodies, bringing together experts from Europol, Interpol, national data protection authorities, academia, forensic laboratories, and civil society, could perform ongoing technological monitoring, identify emerging risks, and formulate operational recommendations before problematic practices become rooted [98].

Over the next years, priorities may be clustered into three sequential phases. In the short term, European data protection authorities should issue explicit guidance on the forensic use of DTC genetic databases, while France should launch a national public consultation on the legal framework applicable to IGG and establish an interministerial working group [98].

In the medium term, the priority should shift toward harmonization and validation. This would include the development of a more explicit European regulatory framework for IGG, the creation of inter-laboratory validation programs for kinship inference tools and DNA mixture deconvolution platforms, and the launch of dedicated research initiatives focused on algorithmic bias, model transparency, and viability in forensic AI systems [99].

In the longer term, more structural steps should be pursued. These could include the creation of a pilot European forensic database based on an opt-in model, the drafting of an additional protocol to the Oviedo Convention addressing specifically the forensic use of genomic data, and the establishment of a certification mechanism for AI systems used in forensic genetics [79].

## 7. Conclusions

### 7.1. What Is Possible Now, and What Requires Caution

This review supports a nuanced distinction between what is already technically viable and, in some jurisdictions, legally operable, and what still requires further validation, tighter regulation, or sustained ethical scrutiny.

Among the practices that can already be considered operational under clearly defined conditions are the controlled use of FIGG for the investigation of serious violent crimes in jurisdictions with an explicit legal framework and an opt-in access model; the deployment of probabilistic STR-based mixture deconvolution software whose scientific validation and judicial acceptance are relatively well established; the limited use of DNA phenotyping for narrowly defined traits such as eye and hair color in legal systems that expressly authorize such applications; the exchange of STR profiles between European forensic laboratories within the Prüm framework; and the implementation of audit and traceability practices across the forensic analytical chain [100,101]. By contrast, several developments require particularly close supervision and stricter safeguards. These include the use of FIGG in jurisdictions that lack a specific legal basis; morphological phenotyping and fine-scale ancestry inference; deep learning models for complex mixture deconvolution that have not yet undergone sufficient independent validation in real-world forensic conditions; the gradual expansion of the purposes for which DTC genetic databases may be accessed; and international transfers of genomic data to third countries that do not offer an equivalent level of legal protection. In these areas, technical capability should not be mistaken for normative legitimacy [3,12,92,102].

A central conclusion of this review is therefore that forensic innovation cannot be assessed solely in terms of performance. In this field, operational usefulness, scientific validity, legal admissibility, and ethical acceptability must all be achieved simultaneously.

### 7.2. Toward a Technical–Legal–Ethical Alliance

The long-term viability of the forensic use of DTC genetic databases depends on a structured alliance among three domains of expertise that still too often operate in parallel rather than in concert. The technical domain must deliver tools that are analytically viable, transparent, documented, and as free as possible from unjustified bias. The legal domain must provide a framework that is clear, proportionate, procedurally fair, and at least partially harmonized across jurisdictions. The ethical domain must ensure that dignity, privacy, equality, and democratic accountability are not subordinated to the sole pursuit of investigative efficiency [9,14,35,45].

This three-way alliance is becoming increasingly urgent because technological evolution is proceeding faster than normative adaptation. Third-generation sequencing, portable genomics, metagenomic approaches, and emerging forms of generative AI applied to biological data may soon alter the practical boundaries of forensic investigation once again. Without shared anticipatory governance, legal systems will remain trapped in a reactive posture, addressing controversies only after the underlying practices have already become entrenched.

Ultimately, the convergence of DTC genetic databases, artificial intelligence, and forensic genetics raises a question that is not merely technical and not merely legal. It is a political and societal question about the type of collective order democratic societies are prepared to accept. A society in which the DNA of millions of citizens can become, without clear awareness or meaningful control, an indirect resource for judicial use is fundamentally different from one in which genomic resources are mobilized under strict conditions of necessity, proportionality, transparency, and accountability to serve the investigation of the most serious crimes [19,98,103].

The distinction between these two trajectories should guide the next generation of forensic governance. Investigative genetic genealogy and AI-assisted forensic tools should not be rejected outright, but neither should they be normalized by technological momentum alone. Their legitimacy will depend on preserving a clear boundary between investigative intelligence and judicial proof, between public-interest use and commercial opportunism, and between scientific innovation and democratic control.

The decisive question is therefore not whether these technologies can expand forensic power, but whether democratic societies can discipline that power without surrendering the rights they seek to protect.

## Data Availability

No new data were created.

## Abbreviations

The following abbreviations are used in this manuscript:

AI	Artificial intelligence
cM	Centimorgan
CCNE	Comité consultatif national d’éthique pour les sciences de la vie et de la santé
CNIL	Commission nationale de l’informatique et des libertés
ddPCR	droplet digital PCR
DTC	Direct-to-consumer
EAFS	European Academy of Forensic Science
ENFSI	European Network of Forensic Science Institutes
FIGG	Forensic Investigative genetic genealogy
FNAEG	Fichier national automatisé des empreintes génétiques
IBD	Identical by descent
NGS	Next-generation sequencing
PCR	Polymerase chain reaction
SNP	Single-nucleotide polymorphism
STR	Short tandem repeat

## Appendix A

### European Countries Without Legislation Specifically and Exclusively Dedicated to Forensic DNA Databases or DNA Profiling (As of 1 January 2026)

This appendix provides a comparative overview of European jurisdictions lacking legislation specifically and exclusively dedicated to forensic DNA databases or DNA profiling as of 1 January 2026, thereby illustrating the regulatory fragmentation that persists across Europe.

A key preliminary distinction must be made: the ENFSI (European Network of Forensic Science Institutes) explicitly recommends that every European country have specific legislation for the implementation and management of its forensic DNA database, as relying solely on the GDPR or Directive (EU) 2016/680 is insufficient to properly regulate these databases [104].

Tree main situations are identified:

**Table A1:** Countries with No Operational National Forensic DNA Database (and therefore no dedicated legislation). These countries had not yet established a functional national database.

Country	Legal Framework Used (Non-Dedicated)
**Moldova**	no forensic DNA database confirmed at the time of the 2024 ENFSI surveys.
**Norway**	no elimination DNA database confirmed, according to responses to the 2024 ENFSI surveys.
**Western Balkans**	forensic institutions from Bosnia-Herzegovina, Serbia, Montenegro, and North Macedonia launched activities to comply with ENFSI recommendations, but without an operational national database or specific dedicated legislation.
**Kosovo**	Internal police presidential directive—a regulatory internal instrument, not a dedicated DNA law.
**Albania**	In the process of aligning with European standards as part of its EU candidacy, with no confirmed specific law.

**Table A2:** Countries with a DNA Database but No Law Exclusively Dedicated to Forensic DNA—General Law or Code of Criminal Procedure Serves as the Framework. Several European countries regulate DNA profiling not through a law specifically and exclusively dedicated to forensic DNA, but through provisions inserted into general legislation (code of criminal procedure, police law, data protection law).

Country	Legal Framework Used (Non-Dedicated)
**Germany**	Bundesdatenschutzgesetz (2015) and Bundeskriminalamtgesetz (BKAG, 2018)—data protection and BKA laws, not a standalone DNA law.
**Poland**	Ustawa o Policji (Police Act, 1990, arts. 20 and 21) and Ministerial Decree (2020)—no standalone DNA law.
**Finland**	Laki poliisin henkilötietojen käsittelystä (Act on the Processing of Personal Data by the Police, 616/2019)—a general police data provision, not a dedicated DNA law.
**Czech Republic**	Internal police presidential directive—a regulatory internal instrument, not a dedicated DNA law.
**Turkey**	Provisions embedded in the Code of Criminal Procedure, with no standalone DNA law.
**Iceland, Liechtenstein**	No documented standalone forensic DNA legislation as of this date.

**Table A3:** Countries with a Law Specifically Dedicated to Forensic DNA (for comparison). The following countries do have dedicated legislation.

Country	Dedicated DNA Law
**United Kingdom**	Protection of Freedoms Act 2012
**France**	Loi relative au FNAEG (2003, amended)
**Spain**	Ley 10/2007
**Netherlands**	Wet DNA-onderzoek bij veroordeelden (2003)
**Belgium**	Loi relative à l’analyse d’ADN en matière pénale (1999, amended)
**Austria**	Sicherheitspolizeigesetz—specific DNA provisions
**Switzerland**	Bundesgesetz über die Verwendung von DNA-Profilen (2000, revised)
**Portugal**	Lei No. 5/2008
**Sweden**	Polisdatalagen (2010)—specific DNA database provisions
**Greece**	Law 3842/2010
**Ireland**	Criminal Justice (Forensic Evidence and DNA Database System) Act 2014

Methodological note:
As of the date the latest ENFSI guidelines were approved (October 2023), not all ENFSI member countries had yet established a DNA database, and consequently not all had dedicated legislation in place. Precise country-by-country data is not always publicly available, and some states (such as Hungary) have refused to share information, making it difficult to produce a fully exhaustive list with absolute certainty.
